# Latin American Internet Survey for Men who have Sex with Men (LAMIS-2018): Design, methods and implementation

**DOI:** 10.1371/journal.pone.0277518

**Published:** 2022-11-17

**Authors:** Michael Reyes-Díaz, Ana Celly, Cinta Folch, Nicolas Lorente, Valeria Stuardo, Maria Amelia Veras, Henrique Barros, Paula Meireles, Dorian Ramírez, Kai J. Jonas, Ulrich Marcus, Axel J. Schmidt, Carlos F. Caceres, Jordi Casabona

**Affiliations:** 1 Centro de Investigación Interdisciplinaria en Sexualidad, Sida y Sociedad (CIISSS), Universidad Peruana Cayetano Heredia, San Martín de Porres, Peru; 2 Universidad Autónoma de Barcelona, Barcelona, Spain; 3 Centre d’Estudis Epidemiològics sobre les Infeccions de Transmissió Sexual i Sida de Catalunya (CEEISCAT), Madrid, Spain; 4 CIBER Epidemiología y Salud Pública (CIBERESP), Madrid, Spain; 5 Coalition PLUS; 6 Instituto de Salud Pública, Universidad Andrés Bello, Santiago, Chile; 7 Faculdade de Ciências Médicas da Santa Casa de São Paulo, São Paulo, Brazil; 8 EPIUnit—Instituto de Saúde Pública, Universidade do Porto, Rua das Taipas, Porto, Portugal; 9 Laboratório para a Investigação Integrativa e Translacional em Saúde Populacional (ITR), Universidade do Porto, Rua das Taipas, Porto, Portugal; 10 Universidad de San Carlos, Guatemala, Guatemala; 11 Faculty of Psychology and Neuroscience, Maastricht University, Maastricht, Netherlands; 12 Instituto Robert Koch, Berlin, Germany; 13 Sigma Research, Department of Public Health, Environments & Society, London School of Hygiene and Tropical Medicine, London, United Kingdom; National Institute of Public Health: Instituto Nacional de Salud Publica, MEXICO

## Abstract

Despite men-who-have-sex-with-men (MSM) from Latin America (LA) are still a vulnerable population for known health-related conditions and social problems, availability of comparable data across LA countries for assessment and monitoring purposes is limited. The objective of this article is to present the study design and the questionnaire of LAMIS-2018 (Latin America MSM Internet Survey), its recruitment strategy, rates and sources by country, and the lessons learned from its implementation. LAMIS-2018 was a cross-sectional, internet-based survey targeting MSM living in 18 LA countries (Argentina, Bolivia, Brazil, Chile, Colombia, Costa Rica, Ecuador, El Salvador, Guatemala, Honduras, Mexico, Nicaragua, Panama, Paraguay, Peru, Suriname, Uruguay, and Venezuela) that gathered data about sexual behaviors, HIV/STI and viral hepatitis knowledge, prophylactic use of antiretrovirals, psychosocial health, and access to sexual health services. The survey went online for four months and was available in three languages (Spanish, Portuguese, and Dutch). Promotion was carried out using dating apps, websites, social networks, and by community-based and academic organizations of each participating country directly in gay venues and in their own premises. Overall, 64,655 MSM participated in LAMIS-2018. Dating apps and websites were the most important recruitment source in most countries, except for Honduras, Nicaragua, and Suriname, where community-based organizations recruited most of the participants. Beyond the LAMIS-2018 implementation description, we highlight the feasibility of such a study in this context, based on the collaboration between community-based and academic organizations to obtain a large sample of MSM in the region. LAMIS-2018 data will contribute to identify determinants of risk behaviors and prevention needs of vulnerable MSM populations in each country of the region.

## 1. Introduction

Men-who-have-sex-with-men (MSM) are a vulnerable population with a high burden of medical and psychological conditions related to negative social experiences that affect their access to proper care, and quality of life [1, 2].

They are considered a key population for the HIV epidemic, reflected in a concentrated and high HIV prevalence (exceeding 10% in nine Latin American countries) and incidence [3, 4]. Besides HIV, MSM also have a higher prevalence of other sexually transmitted infections (STI) such as syphilis, gonorrhea, chlamydia [5], human papilloma virus [6], and hepatitis B virus [7], and enteric diseases related to oral-anal sexual behavior, such as Hepatitis A [8, 9], and blood-borne infections such as hepatitis C.

In addition, studies from Brazil and Mexico reported higher percentages of poor mental health among, including MSM depression, anxiety [10, 11], substance and alcohol abuse [11], and frequent sex alcohol and drug use (28%) [12], the latter being associated with riskier sexual behavior, depression and drug abuse [13].

MSM also faced structural (including demographic, social, economic, political, and cultural) barriers, considered the most relevant determinants of their increased vulnerability in LA [14]. Sex and gender-related violence, homophobia, stigma and discrimination have been frequently reported in LA countries such as Brazil [15], Mexico [16], Chile [17], Peru [18] and in Central America [19], which hinder their access to comprehensive HIV/STI preventive measures and care [14, 20, 21]. They are associated with increased psychological distress, depressive symptoms, and unhealthy practices [19, 22], including risky sex and lower adherence to antiretroviral treatment [20]. It has been shown that in several LA countries, lower socioeconomic status (SES) is associated with lower condom use [23], less knowledge about HIV transmission and pre-exposure prophylaxis (PrEP) [24, 25], less testing and awareness of HIV serostatus [26] but more self-reported positive HIV status [27].

Reliable multidisciplinary surveillance data among MSM is essential, but its availability is still limited in LA, not comparable across different territories or lacking periodicity.

Most multi-country available data focus to monitor the HIV epidemic. In this regard, the Joint United Nations Programme on HIV/AIDS (UNAIDS) have developed the Key Population Atlas (https://kpatlas.unaids.org/dashboard), which aims to collect and share country-level HIV-related data among key populations [28]. However, information from some indicators are missing in some countries since they are not measured or reported by their national health systems.

No similar data sources have been found to collect country-level population-based data regarding mental health or structural disparities among MSM, depending only on academic publications or community-based reports. However, academic publications are also highly focused on HIV/STI-related topics. Even among the HIV literature, there is a huge gap in the availability of strategic data on determinants of access to prevention and care, and the impact of programs geared to these populations in LA [29]. Robust information on behaviors and needs of MSM in LA is currently available but only for a few larger countries, mainly Brazil, Peru and Mexico [30, 31], and they use different approaches (designs, recruitment methods, data collection strategies and instruments, analysis techniques, indicators, etc.) that limit comparability across countries or even regions within the same country.

In recent years, the Internet has become an important setting for recruiting large samples of MSM in HIV research [32, 33]. Online recruitment represent a useful alternative approach to reach an important and often hidden group of MSM from different LA countries [34, 35], and collect relevant information on a wide range of topics in a single cross-sectional questionnaire [36, 37].

The Ibero-American Network of Studies on Gay Men, other MSM and Transgender People (RIGHT PLUS, from its Spanish abbreviation) is a network of both academic groups and NGOs from LA, Spain and Portugal currently part of the Coalition Plus organization (http://right-plus.ceeiscat.cat). It aims at facilitating, implementing and disseminating collaborative participatory research among vulnerable populations in Ibero-America. RIGH Plus in collaboration with researchers from the European MSM Internet Survey (EMIS-2017) project (www.emis2017.eu) [38] implemented the Latin American Internet Survey for Men who have Sex with Men (LAMIS-2018) simultaneously in 18 LA countries (Argentina, Bolivia, Brazil, Chile, Colombia, Costa Rica, Ecuador, El Salvador, Guatemala, Honduras, Mexico, Nicaragua, Panama, Paraguay, Peru, Suriname, Uruguay, and Venezuela). The main purpose of the LAMIS-2018 survey was to collect information on sexual behavior, HIV knowledge, risk exposure, prophylactic use of antiretroviral treatments (PEP and PrEP), psychosocial health and access to sexual health services, among MSM from the LA region in a consistent manner with the EMIS 2017 survey. These data are crucial to better understand the determinants of transmission, prevention and care, and therefore to improve prevention and control policies for HIV and other health and social problems.

Giving the lack of information regarding implementation experiences and challenges using internet-based studies in MSM, the objective of this article is to describe the methodological approach used in LAMIS-2018: the various recruitment strategies used, and the recruitment rates by country. This represents a double opportunity to evaluate the feasibility of such a study, based on collaboration between academic organizations to obtain a large sample of MSM in the region, and to discuss its contribution to sexual health prevention policies in the LA Region.

## 2. Methods

### 2.1. Study design

LAMIS was a cross-sectional, internet-based survey targeting MSM living in 18 LA countries (Argentina, Bolivia, Brazil, Chile, Colombia, Costa Rica, Ecuador, El Salvador, Guatemala, Honduras, Mexico, Nicaragua, Panama, Paraguay, Peru, Suriname, Uruguay, and Venezuela) and was available in three languages (Spanish, Portuguese, and Dutch).

No sample size was calculated for LAMIS-2018 as we are using convenience sampling, and we also aimed to describe the number of responses obtained during the 4-month study period, giving the different recruitment strategies employed.

### 2.2. Inclusion and exclusion criteria

LAMIS-2018 inclusion criteria were 1) self-identify as a men or transgender men who is sexually attracted to men and/or who have ever engaged in sexual interaction with other men, 2) 18 years old or older, 3) who resided in any of the 18 participating LA countries at the time of the survey, and 4) indicating that they understood the nature and purpose of the study and that they consented to take part in it. There were not additional exclusion criteria than not meeting inclusion criteria.

### 2.3. Questionnaire design

The LAMIS-2018 questionnaire was programmed on the Demographix online platform. It consists of slightly adapted versions of the Spanish, Portuguese and Dutch language versions used for EMIS-2017, which was conducted between 2017 and 2018 in 46 European countries, plus Lebanon, Israel, Canada, and the Philippines, and was available in 33 languages [38].

The version used in LAMIS went through peer review by the RIGHT PLUS members and was piloted in all participating countries to ensure overall clarity and interpretability.

The questionnaire covered different topics which would be later grouped in corresponding theme chapters:

1. Sociodemographic, identity and sexuality characteristics: including information about age, sex assigned at birth and gender identity, place and size of residence, educational level, employment status, financial coping, migration and origin, relationship status and transactional sex.
2. Psycho-socio-sexual health status: including information regarding depression and anxiety symptoms, sexual happiness, alcohol dependency, HIV diagnosis and viral suppression, diagnosed STIs (Syphilis, gonorrhea, chlamydia, human papillomavirus), history of hepatitis B and C.
3. Risk and precaution behavior: including information regarding age at first sex and anal intercourse, number of steady male, non-steady male, and female sexual partners, condom use, recreational, sexualized and injecting drug use, use of HIV pre-exposure prophylaxis (PrEP) and post-exposure prophylaxis (PEP); HIV treatment uptake, hepatitis A and B vaccination coverage, and detailed information about last sexual encounter with male non-steady sexual partners.
4. Social and health needs: including information about social support, internalized homonegativity, concerns about alcohol and other drug consumption; condom use self-efficacy, knowledge about HIV transmission and preventive strategies, access and barriers to PEP, PrEP and HIV antiretroviral treatment (ART), and knowledge on STI transmission, on PEP, PrEP, and on viral hepatitis.
5. Offer and use of health services: including information about usage of services for drug and alcohol dependency, access to condoms, access to HIV/STI information, access to PrEP information, prescriptions and pills, access to HIV testing and care services, access to STI testing, partner notification for STI treatment, and offer of hepatitis vaccination.

The LAMIS questionnaire differed from the EMIS-2017 questionnaire [38], in the following aspects: using educational levels typical for LA countries instead of the years spent in education after age 16; collection of data on homonegativity and social support scales from all participants (rather than from a randomized sub-sample as in EMIS-2017); adaptation of the questions on belonging to an ethnic minority (changed to belonging to a indigenous ethnic group, being of African descent, or belonging to a native community).

The final online version of the questionnaire had 35 core pages (62 questions) and 20 additional questions that were shown when a condition question was answered. The first page of the questionnaire constituted the introduction to LAMIS-2018 and indicated the inclusion criteria to be met by each participant. The questionnaire itself started at the second page.

### 2.4. Promotion and recruitment sources

Community-based organizations from each participant country were identified and invited as local partners to articulate promotion and recruitment activities in their territories. From January 24th to May 13th, 2018 (110 days), LAMIS-2018 was promoted through MSM dating apps, websites, social media, clinics, and gay men’s organizations or venues from the 18 LA countries.

**Fig 1:**
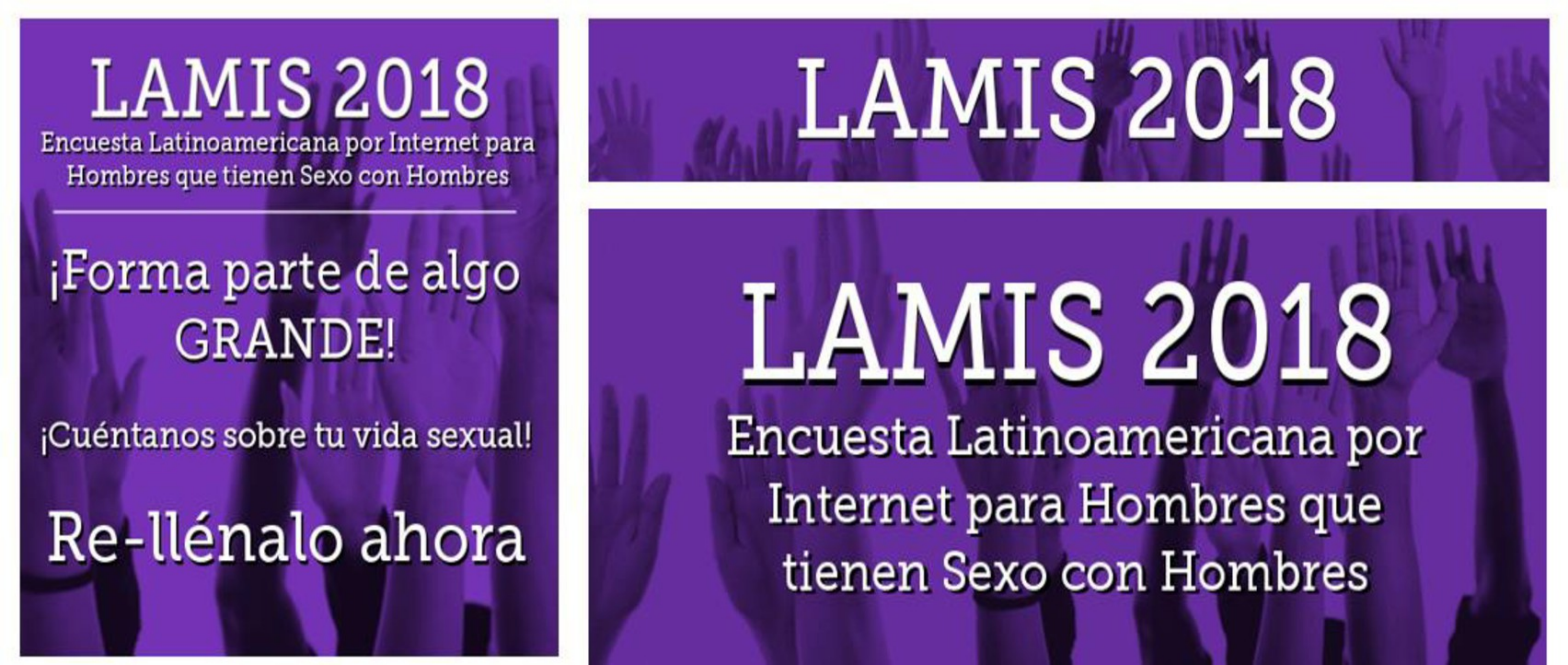
Examples of banners for the promotion of the LAMIS-18 survey.

The online promotion had different images, banners, and material in the three languages of the study (Fig 1). The design used for the EMIS survey promotion was maintained but using a violet tone instead of blue to represent the LAMIS study. Offline (non-virtual) promotion was also carried out using printed materials (i.e., posters and cards) placed or distributed in gay entertainment venues (e.g., discotheques, saunas, etc.).

Recruitment sources for the LAMIS study were:

- LAMIS-2018 website was created and the link was shared though direct messages.
- Websites and dating apps for MSM, in particular Grindr, PlanetRomeo, Hornet. Those sent out direct messages to their users. Grinder posted six 24-hour messages in its app, strategically distributed throughout the recruitment period. PlanetRomeo broadcasted an instant message at the beginning of the recruitment period. Manhunt and Jack’d published banners in Spanish and Portuguese on their websites and in their application for mobile phones on three occasions. Hornet participated in a massive campaign at the beginning and end of the recruitment. Recruitment via websites and dating apps was arranged by the co-author at the Robert Koch Institute (Berlin), Grindr and PlanetRomeo offered their services free of charge. Additional funding was available to pay for extra promotion in Brazil using Hornet.
- Most popular social networks and web pages such as Facebook, Instagram, Twitter, and Youtube were also used, through collaborative partners´ institutional accounts.
- Local dissemination by the various collaborating organizations (NGOs or community-based LGBTIQ or HIV/AIDS organizations, activists, research groups, etc.) placing banners on their websites, sending invitation messages to their users through their mailing lists, sending news to digital magazines, collecting participants’ answers during outreach activities, etc.

### 2.5. Screening, enrollment and data collection

People reached by recruitment strategies gained access to a link that directed them to the survey web page. The first page contained the informed consent providing study information, including a brief summary of the study purpose and its relevance, institutions involved, inclusion and exclusion criteria, benefits and potential harms, estimated time to complete the survey, and data usage. Potential participants were also told that the study won’t save any identifying information from them or their servers such as TP/IC address or cookies, thus if they left the web page without completing the survey, it wouldn’t be possible to go back to continue where they left and information would be lost. Before moving on to the survey questions, all participants must have stated that they understood the nature and purpose of the study and that they consented to take part in it. After consent, the only compulsory questions were the first ones about age, sexual identity, sex assigned at birth and country of residence, to verify inclusion and exclusion criteria. The Demographix online platform only saved data from completed questionnaires, meaning those who reached to the ending screen of the questionnaire.

### 2.6. Analysis plan

We calculated descriptive data about attrition, recruitment rate and recruitment source of information, using percentage presented in tables, stratified by survey language version or country.

Attrition rate was defined as the percentage of people who had access to the survey but withdrew at a certain point among the total amount of people who have accessed to it. Three attrition rates will be presented: 1) Total withdrawal from first page, as withdrawal at any point along the questionnaire among all people who reached the first page 2) Withdrawal before consent, as withdrawal between first (information and consent page) and second page (where questionnaire’s questions start) among people who reached the first page; and 3) Withdrawal after consent, as withdrawal at any point along the questionnaire among people who reached the second page (where survey’s questions start). Attrition information was calculated among all potential participants and stratified by the three questionnaire language versions.

Recruitment rate was defined as the percentage of LAMIS-2018 valid responses over the number of male population between 15 and 65 years of age according to national census data until 2017. Recruitment information was calculated among the whole sample and stratified by country.

### 2.7. Ethical approval

For the regional implementation of the LAMIS study, the ethical approval of the Ethics Review Committees of the Universidad Peruana Cayetano Heredia (Lima), the Faculty of Medicine, University of Chile (Santiago), the Santa Casa de Misericórdia (São Paulo) the National Committee for Health Ethics (Guatemala), and the Faculty of Psychology and Neuroscience of the University of Maastricht (The Netherlands), were obtained.

According to preferred language by a potential participant, approved online written informed consent was available in Spanish, Portuguese or Dutch, in the first page of the questionnaire, before accessing the survey questions.

## 3. Results

### 3.1. Attrition rate

During recruitment period, 163.439 people accessed the LAMIS-2018 online questionnaire. Among them, the Demographix platform reported that 93,632 people (57.6%) abandoned the questionnaire at any point; this was higher among the Dutch version recruits (74.9%). Overall, 33.7% of recruits abandoned the questionnaire immediately after the first page (also higher in the Dutch version with 57.3%). The dropout rate from the second page (where the questionnaire started after confirming inclusion criteria) until the end of the questionnaire was 36.1% in the entire sample (Table 1). Dropouts from seven additional cases were not reported by the online server. A total of 68,800 people finished the survey.

**Table 1:** Attrition rates throughout LAMIS-2018 questionnaire by language version.

Attrition rates	Spanish version		Portuguese version		Dutch version		Total	
	n	%	n	%	n	%	n	%
People who reached first page [A]	114.179		47.332		928		162.439	
People who reached second page* [B]	77.217		30.027		396		107.640	
People who reach final page [C]**	49.261		19.313		233		68.807	
**Total withdrawal from first page [(A-C)/A]**	64.918	56,9%	28.019	59,2%	695	74,9%	93.632	57,6%
**Withdrawal before consent ǂ [(A-B)/A]**	36.962	32,4%	17.305	36,6%	532	57,3%	54.799	33,7%
**Withdrawal after consent ǂǂ [(B-C)/B]**	27.956	36%	10.714	36%	163	41%	38.833	36%

*After consenting participation

** The Demopraphix platform reported dropouts from 93.632 people, missing 7 dropout cases.

**ǂ** Withdrawal between first and second page

**ǂǂ** Total withdrawal of those who reached the second page

### 3.2. Total returns and data cleaning process

**Fig 2:**
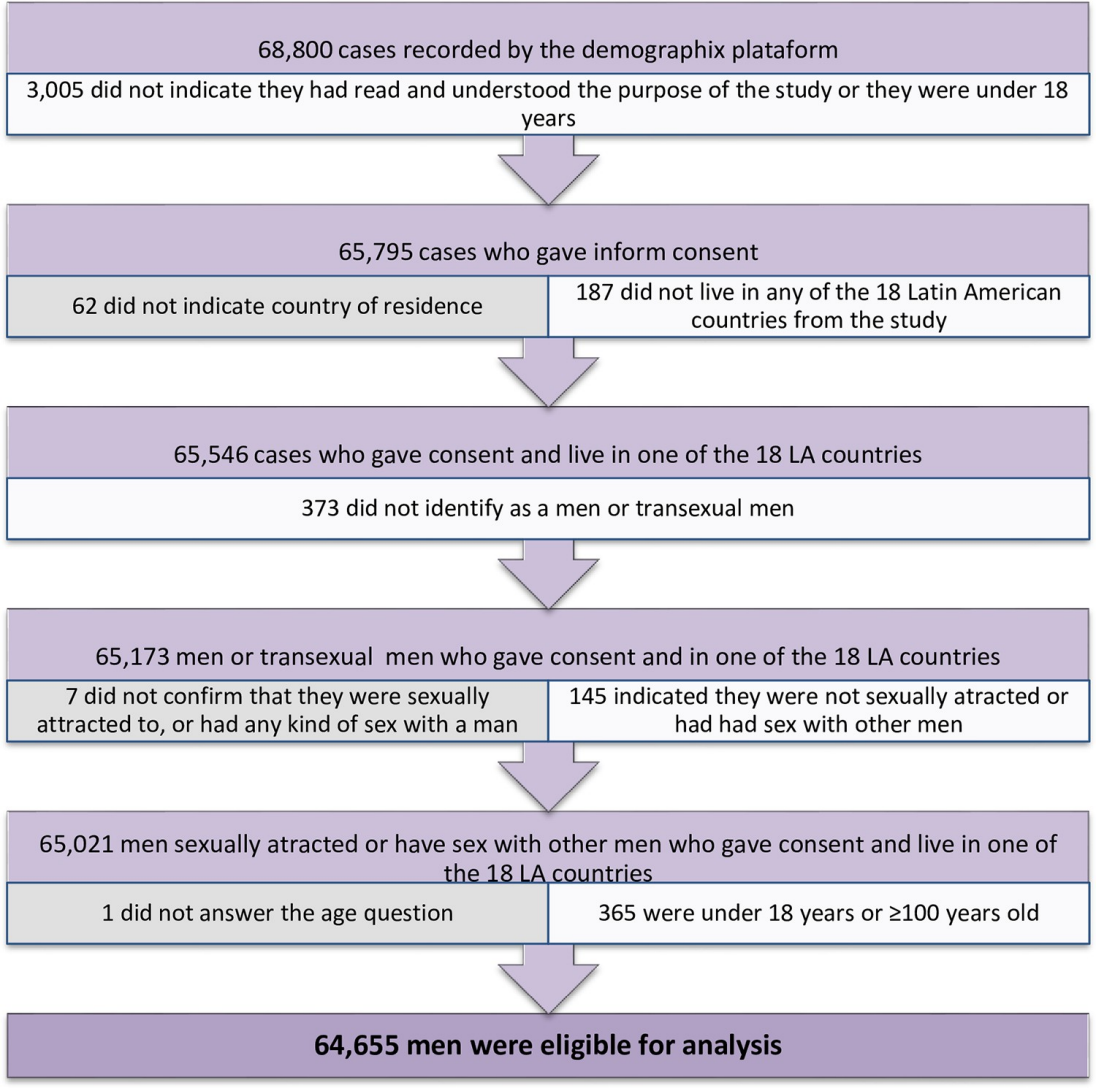
Data cleaning process from cases recorded online to final valid questionnaires.

After closing the data collection there were 68,800 cases recorded. After data cleaning, 4,145 people who didn’t met inclusion criteria were removed: participants who indicated that they had not read or understood the information on the introductory page, were under 18 years of age, resided in a country other than those included in the study, did not identify as male or transgender male, were not attracted to other men, or did never have sex with another man, resulting in 94% (64,655) of qualified cases (Fig 2).

### 3.3. Datasets

**Fig 3:**
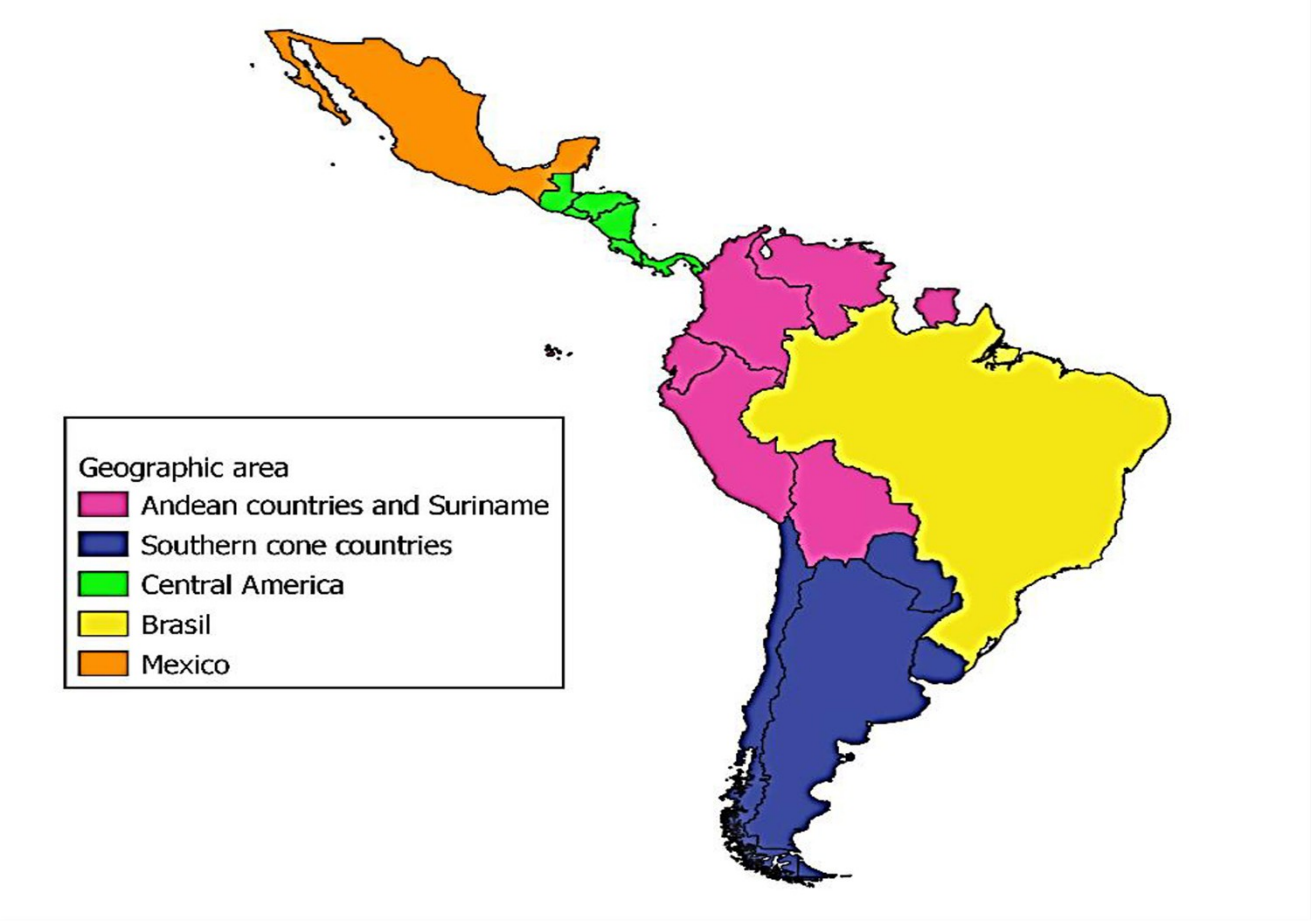
Grouping criteria by geographic area among LAMIS-2018 participant countries from the Latin American region. Geographical areas: 1. Andean countries and Surinam included Surinam and the Andean countries Bolivia, Ecuador, Peru, Colombia and Venezuela. Suriname was included in this group due to its geographic proximity to Venezuela and its small population size. French Guiana was included in EMIS-2017 as a French overseas territory; Guyana was not included due to its anti-gay legislation 2. The southern cone countries include Argentina, Chile, Paraguay and Uruguay, located in the southernmost part of the American continent, which, like a large peninsula, defines the southern part of the South American subcontinent. 3. Central America includes Costa Rica, El Salvador, Guatemala, Honduras, Nicaragua and Panama, located between Mexico and South America. 4. Brazil, given its size and the fact that it is the only Portuguese-speaking country. 5. Mexico, given its size and northeast LA location.

LAMIS-2018 dataset has a total of 64,655 qualified cases. National datasets are available for all 18 countries with 200 or more qualifying cases. In the LAMIS-2018 final report (see the report and executive summary published: http://www.coalitionplus.org/LAMIS/), full data analysis was performed, presenting overall descriptive results, disaggregated by geographic area and/or country if considered necessary. The country-grouping criteria by geographical areas is described in Fig 3.

The LAMIS database is the legal property of the Institut d’Investigacions Biomèdiques Germans Trias i Pujol (IGTP)/ Centre d’Estudis Epidemiològics sobre les Infeccions de Transmissió Sexual i Sida de Catalunya (CEEISCAT). CEEISCAT manages the data and data assignments made to third parties.

### 3.4. Recruitment rate by country

The mean country recruitment rate in LAMIS-2018 was 3.3 participants per 10,000 men (Table 2). The countries with higher recruitment rates (higher than 7 participants per 10,000 men 15 to 65 years of age) were Suriname (11.7) and Chile (8.2). Lower recruitment rates (3 or lower per 10,000 men 15 to 65 years of age) were found in El Salvador (2.8), Ecuador (2.7), Paraguay (2.7), Nicaragua (2.7), Brazil (2.5), Guatemala (2.4), Venezuela (2.3), Honduras (2.3), Bolivia (2.2) and Peru (1.9) (Table 2).

**Table 2:** Recruitment rate in LAMIS-2018 by country.

Country	Men aged 15–65 by national census 2017	LAMIS participants	Recruitment rate per 10,000 men 15–65 years old	% qualifying participants in LAMIS
Brazil	71,955,960	18,139	2.5	28.1%
Mexico	40,812,857	14,957	3.7	23.1%
Colombia	16,364,021	8,208	5.0	12.7%
Argentina	14,031,178	5,504	3.9	8.5%
Chile	6,063,031	4,945	8.2	7.6%
Venezuela	10,417,499	2,431	2.3	3.8%
Peru	10,579,952	2,025	1.9	3.1%
Ecuador	5,243,222	1,440	2.7	2.2%
Guatemala	4,747,391	1,157	2.4	1.8%
Costa Rica	1,700,046	1,012	6.0	1.6%
Uruguay	1,119,302	771	6.9	1.2%
Panamá	1,311,089	759	5.8	1.2%
Bolivia	3,477,538	748	2.2	1.2%
Honduras	2,870,553	646	2.3	1.0%
El Salvador	2,062,035	572	2.8	0.9%
Paraguay	2,172,676	591	2.7	0.9%
Nicaragua	2,004,397	534	2.7	0.8%
Suriname	184,261	216	11.7	0.3%
Total	197,117,006	64,655	3.3	100.0%

The countries with the largest numbers of participants are Brazil (28.1%), Mexico (23.1%) and Colombia (12.7%) contributing 63.9% of all participants, reflection overall population sizes of participating countries (65%).

**Fig 4:**
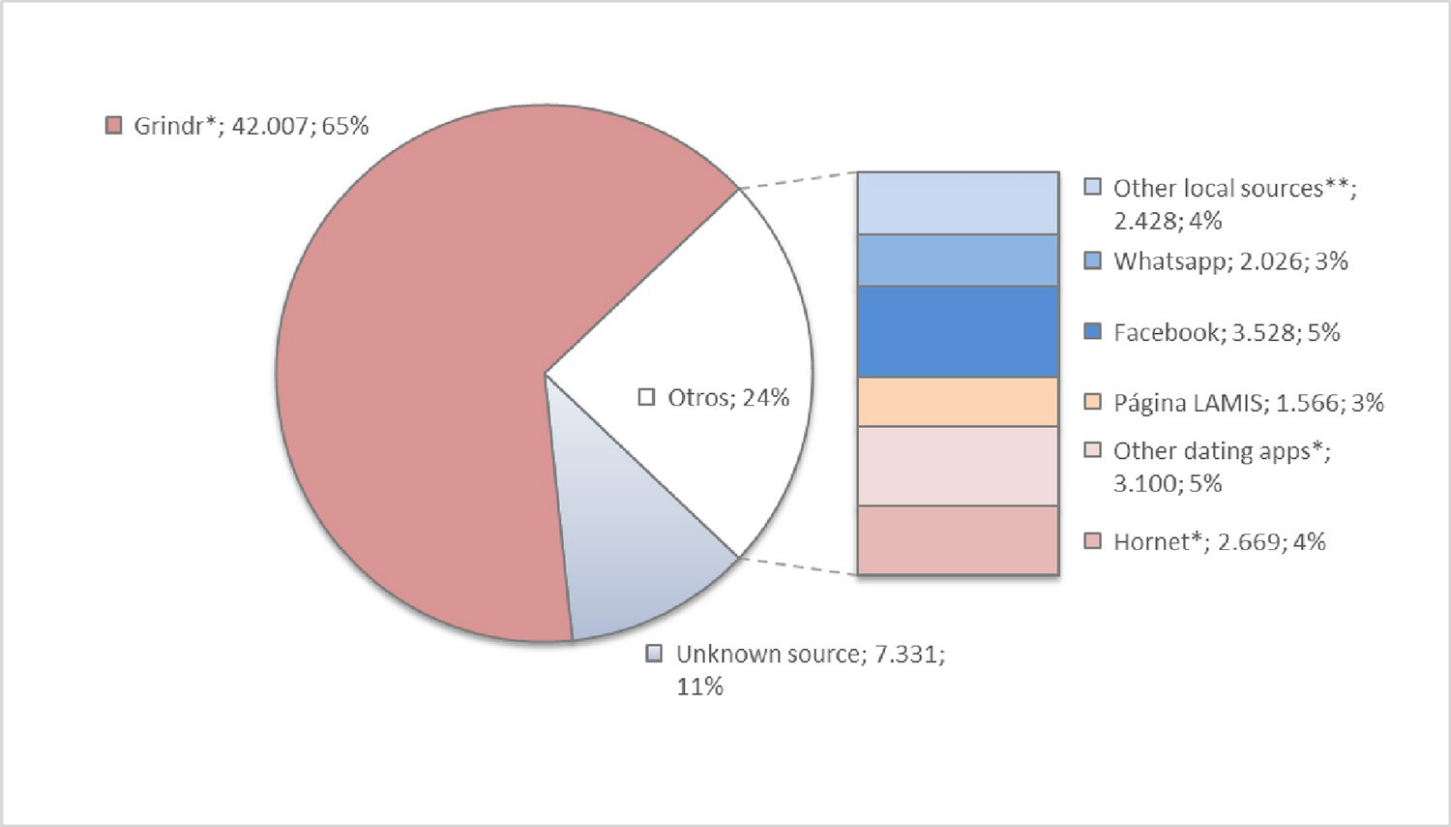
Recruitment sources used in LAMIS-2018. *Centrally arranged by co-author at the RKI Institute (Berlin) **Local dissemination by the various collaborating organizations (NGOs or community-based LGBTIQ or HIV/AIDS organizations, activists, research groups, etc.).

As shown in Fig 4, Grindr obtained the highest proportion of participants, representing the 65% of the whole study with Colombia and Argentina being the countries with the highest recruitment through this tool with 83% and 81% respectively, while Suriname had the lowest proportion with only 14% (Table 3).

**Table 3:** Recruitment sources used in LAMIS-2018 by country.

Country	Grindr*	Hornet*	Other dating apps*	LAMIS Page	Facebook	WhatsApp	Other local sources**	Unknown source
Argentina	80.6%	0.3%	9.4%	0.8%	0.7%	0.1%	5.7%	2.5%
Bolivia	51.1%	0.1%	1.9%	0.3%	42.0%	0.3%	0.5%	3.9%
Brazil	55.3%	10.9%	1.5%	3.8%	2.8%	9.8%	3.0%	12.9%
Chile	51.5%	1.9%	2.9%	2.4%	3.2%	1.0%	16.6%	20.5%
Colombia	83.4%	0.3%	4.4%	0.3%	1.3%	1.3%	1.3%	7.7%
Costa Rica	76.0%	1.4%	3.4%	0.0%	7.7%	0.0%	2.3%	9.3%
Ecuador	71.4%	0.6%	3.5%	0.1%	11.0%	2.2%	2.1%	9.0%
El Salvador	50.5%	0.2%	1.6%	0.2%	19.8%	0.0%	15.7%	12.1%
Guatemala	58.3%	0.0%	1.6%	0.4%	30.6%	0.3%	2.3%	6.4%
Honduras	38.5%	0.2%	1.1%	0.3%	50.9%	0.0%	0.6%	8.4%
México	71.6%	3.3%	6.6%	0.3%	0.7%	0.1%	3.0%	14.4%
Nicaragua	35.2%	0.0%	2.1%	0.2%	57.7%	0.4%	0.6%	3.9%
Panamá	69.8%	0.0%	1.8%	0.3%	23.7%	1.3%	1.6%	1.4%
Paraguay	61.4%	0.7%	1.7%	0.2%	28.6%	0.5%	5.4%	1.5%
Peru	63.0%	0.3%	7.8%	0.4%	18.5%	0.2%	3.7%	6.1%
Suriname	13.9%	0.0%	0.9%	0.0%	33.3%	1.4%	39.4%	11.1%
Uruguay	24.4%	4.2%	5.8%	0.6%	16.1%	0.4%	12.5%	36.1%
Venezuela	60.8%	0.2%	18.0%	0.4%	1.9%	0.1%	13.0%	5.6%
Total	65.0%	4.1%	4.8%	1.5%	5.5%	3.1%	4.7%	11.3%

*Centrally arranged by co-author at the RKI Institute (Berlin)

**Local dissemination by the various collaborating organizations (NGOs or community-based LGBTIQ or HIV/AIDS organizations, activists, research groups, etc.)

Hornet was the second most important dating app for recruitment in the study. It accounted for 4% of recruitment (Fig 4). In Brazil, this app was paid for a massive promotional campaign and in this country, it represented 12% of the total recruitment. In the rest of the countries, the percentage of recruitment ranged from 0% (Guatemala, Nicaragua, Panama, and Suriname, among others) to 4% (Uruguay) (Table 3).

Facebook was the social media with the highest recruitment share, representing 5% of the total recruitment (Fig 4). It should be mentioned that, with only a few weeks to go before the end of the promotion, advertising was paid via Facebook to reinforce recruitment in countries with low recruitment rates. Because of this advertising campaign, Facebook represented the most important recruitment source in Central America (Table 3), especially in Nicaragua and Honduras where the proportion of recruited participants was 58% and 51% respectively (Table 3).

Participation through WhatsApp messages accounted for 3.1% of the overall sample (Fig 4), and was especially relevant in Brazil, where it represented 9.8% of the overall recruitment (Table 3); in the rest of the countries, it ranged between 0% in Costa Rica and 2.1% in Ecuador. Sampling in Suriname was mostly done via networks of the local collaboration partner and offline sampling.

## 4. Discussion

LAMIS-2018 survey was successfully carried out, implemented and executed simultaneously in 18 LA countries, targeting MSM, and reached the largest known MSM on-line sample in the region and by country. Similar studies reached smaller sample sizes: the Global Forum on MSM & HIV (MSMGF) in 2012 reached a sample size of 5,779 participants worldwide, with 880 participants from LA countries [39]; Biello *et al*. conducted an online survey in 2012 that reached a total on 29,787 participants from Mexico, South and Central America, but reached a lower per country sample size, i.e. less than 500 participants, in Ecuador, Uruguay, Bolivia, Paraguay, and all Central American countries [36]; a more recent 2018 survey conducted only among MSM from Brazil, Mexico and Peru, reached 19,457 MSM without a self-reported HIV diagnosis, lower than the 29,260 equivalent cases from LAMIS-2018 in those three countries [40]. Smaller countries, like Suriname, that also do not speak Spanish or Portuguese are often not represented at all in such surveys.

We found that about 60% of people who reached the first screen of the survey (informed consent) [Table 1] completed the questionnaire, being comparable with previous studies retention: Biello et al. reported that out of 56,584 people who clicked their survey link, 30,063 completed it (about 64%) [38], and Torres et al. reported that questionnaire completion was 45% [42]. Our completion rate thus falls into the range obtained before. Probable reasons for not completing the survey are reaching people online who do not fulfill inclusion criteria, lack of interest in participation, perception that the questionnaire is taking a lot of time, internet connection problems, and fear of sexual identity disclosure if being discovered while filling in the survey. We couldn’t assess these reasons as data from drop-outs was erased, but partners from Suriname reported that fear of sexual identity disclosure was an important factor.

Benefiting from EMIS experience and resources, and the joint work with local LA research groups and NGOs, LAMIS-2018 executed a mixture of recruitment strategies, adapted to specific settings and contexts. Free Grindr promotion was very important, mainly using personal invitations / direct per user messages, accounting for 42,000 valid questionnaires (65% of the total). Other dating apps provided less participants, except for some of them in specific countries (e.g., Hornet in Brazil). In general, MSM dating apps were less popular in Honduras, Nicaragua, and Suriname, where local collaborating organizations (NGOs, community-based LGBTIQ or HIV/AIDS organizations, activists, research groups, etc.) brought the majority of participants (52%, 59%, and 74% respectively). In the case of some Central American (Honduras and Nicaragua) and other South American countries (Bolivia and Paraguay), unplanned paid Facebook promotion was used to increase recruitment rates. Cases such as the use of WhatsApp in Brazil, the only country where it yielded a significant proportion of participants, paves the way to increase its use as a source of recruitment in future studies.

Two principal sources of bias were identified in LAMIS-2018: Uneven access to the internet across countries, and self-selection bias in the recruitment process [38, 41]. As LAMIS-2018 was a study conducted using an online questionnaire, this was completed by an audience with access to mobile or desktop devices, and the internet. In addition, the promotion was focused on online dating apps, social media and web pages frequently visited by MSM, highlighting in importance the recruitment by applications for dating and encounters between men, characteristics that imply a possible lower representation of those MSM populations with fewer resources, residents of areas with little internet access, less integrated into the LGTBIQ community, and who do not frequently use these applications for dating and encounters with other men. Studies comparing internet- vs field-sampling surveys from the UK and China have previously reported that internet-based studies reached younger MSM, with higher education or socio-economic status, reporting higher risk behavior, higher HIV testing [42] and more commonly identifying as ‘gay’ [43].

Besides its limitations, LAMIS-2018 has proven the strength of conducting an online study in the LA region among the MSM population, as a feasible and, given the free promotion by Grindr and PlanetRomeo, a relatively low-cost alternative to face-to-face surveys. This project has also proved that online studies are a powerful tool to update and fill the gap of information concerning behavioral and psycho-socio-sexual health-related aspects for planning and monitoring prevention programs for MSM, which has been a constant issue in the LA region. In addition, LAMIS-2018 reached a large sample size, sufficient to perform most descriptive and comparative analysis by country or in main cities, and to ensure statistical power when performing hypothesis testing.

LAMIS-2018 contribution to science is not only related to the data obtained, but also to the network established through the implementation of this study. Researchers from different countries were able to work together and form the RIGHT PLUS network for future academic and research collaborations in the Latin-American region. With a participatory research approach in mind, the project reinforced the crucial role that community-based organizations should play in the design and implementation of research on MSM, in collaboration with academic and research institutions.

In addition, LAMIS-2018 responded to the lack of information regarding implementation experiences from internet-based research targeting LA MSM. Sharing this experience will allow the adoption of successful strategies and improvement of the less effective ones by future research and community efforts using internet-based recruitment and data collection tools.

Finally, LAMIS will be the basis for other studies strengthening community work, identifying socio-demographic characteristics, determinants of risk behaviors, and prevention needs of vulnerable MSM populations in each country; in particular, the replication of the survey in the future will also allow for assessing the evolution of these aspects overt time in the region.
